# Fighting bacterial pathogens with carbon nanotubes: focused review of recent progress

**DOI:** 10.1039/d3ra01745a

**Published:** 2023-06-29

**Authors:** Mihaela Asaftei, Massimiliano Lucidi, Cristina Cirtoaje, Alina-Maria Holban, Costas A. Charitidis, Fang Yang, Aiguo Wu, George A. Stanciu, Özge Sağlam, Veronica Lazar, Paolo Visca, Stefan G. Stanciu

**Affiliations:** a Center for Microscopy-Microanalysis and Information Processing, University Politehnica of Bucharest Romania stefan.g.stanciu@upb.ro; b Department of Microbiology, University of Bucharest Romania; c Department of Science, Roma Tre University Rome 00146 Italy paolo.visca@uniroma3.it; d NBFC, National Biodiversity Future Center Palermo 90133 Italy; e Department of Physics, University Politehnica of Bucharest Romania; f Research Lab of Advanced, Composite, Nano-Materials and Nanotechnology, School of Chemical Engineering, National Technical University of Athens Greece; g CIXI Institute for Biomedical Engineering, Ningbo Institute for Materials Technology and Engineering, Chinese Academy of Sciences China; h Department of Mechanical Engineering, İzmir University of Economics Turkey; i Santa Lucia Foundation IRCCS Rome 00179 Italy

## Abstract

The fast and global spread of bacterial resistance to currently available antibiotics results in a great and urgent need for alternative antibacterial agents and therapeutic strategies. Recent studies on the application of nanomaterials as antimicrobial agents have demonstrated their potential for the management of infectious diseases. Among the diverse palette of nanomaterials currently used in biomedical applications, carbon nanotubes (CNTs) have gained massive interest given their many valuable properties, such as high thermal and electrical conductivity, tensile strength, flexibility convenient aspect ratio, and low fabrication costs. All these features are augmented by facile conjugation with functional groups. CNTs are currently available in many configurations, with two main categories being single-walled and multi-walled CNTs, depending on the number of rolled-up single-layer carbon atoms sheets making up the nanostructure. Both classes have been identified over the past years as promising antibacterial agents but the current level of understanding of their efficiency still harbors many pending questions. This mini-review surveys recent progress on the topic of antibacterial effects of CNTs and examines the proposed mechanisms of action(s) of different CNT typologies, placing the main focus on past studies addressing the antibacterial activity on *Staphylococcus aureus* and *Escherichia coli*, two prototypical Gram-positive and Gram-negative pathogens, respectively.

## Abstract 2

Main effects of SWCNTs and MWCNTs on *E. coli* and *S. aureus* bacterial cells.

## Graphical abstract

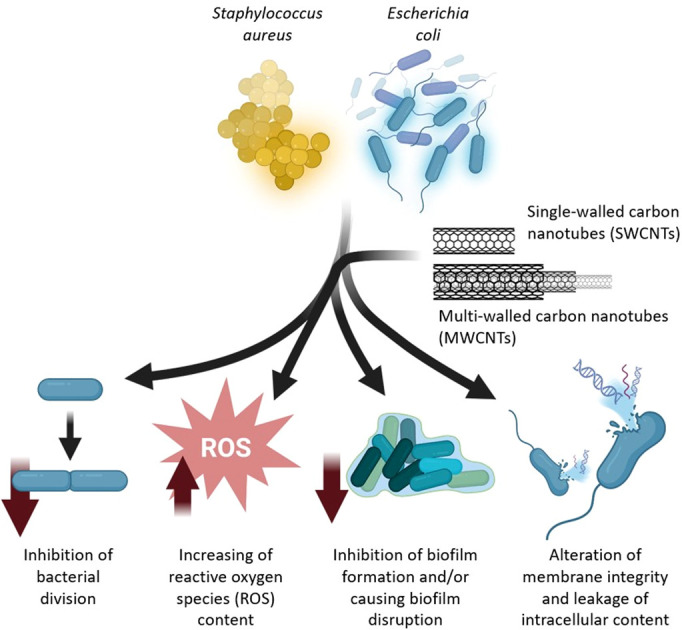

## Introduction

Microorganisms are widely found in soil, water, plants, wild and domestic animals, humans, or foods. While most species are beneficial for supporting current life on Earth, a small number are pathogenic and can cause disease to humans and animals. Many pathogenic microorganisms, especially those circulating in healthcare settings, have developed resistance to most of the available antibiotics and can cause severe infections accounting for tens of millions of deaths annually across the globe.^2,3^ Among the most alarming pathogenic bacterial species, those comprised in the ESKAPE group (*Enterococcus faecium*, *Staphylococcus aureus*, *Klebsiella pneumoniae*, *Acinetobacter baumannii*, *Pseudomonas aeruginosa*, and *Enterobacter* species) have gained the main focus of attention over the past decades.^4^ These species are considered to be the most common bacterial pathogens in healthcare-associated (nosocomial) infections, causing extensive morbidity and mortality, especially in critically ill and immunocompromised patients.^5,6^ ESKAPE pathogens are characterized by a high level of antibiotic resistance,^7^ which recently prompted the World Health Organization to list them among the greatest threats to human health and to encourage research on new effective drugs for the treatment of antibiotic-resistant infections,^8^ which are urgently needed.

The great genetic adaptability, intrinsic bacterial resistance genes, and the selective pressure exerted by the massive use of antibiotics are responsible for the appearance, transfer, and spread of antibiotic resistance genes and bearing strains.^9^ Other factors contributing to the emergence of drug-resistant strains are thoroughly discussed in the recent review of Larsson *et al.*^10^ Recently, nanomaterials emerged as important tools in the fight against multidrug-resistant (MDR) bacteria.^11^ These materials can be used as “nano-weapons” that can act individually or in synergism with antimicrobial compounds against bacteria. This synergism holds valuable intrinsic potential for the development of next-generation, all-in-one agents, that can combat both drug-susceptible and MDR strains. The most common mechanism of action of nanomaterials relies on their interaction with the cellular envelope of bacteria, causing its destabilization and ultimately cell death, even for highly resistant species.^12^ Currently, among the most studied nanomaterials proposed as alternative antibacterial agents, metal-based nanoparticles (NPs), graphene-based nanomaterials, and carbon dots have extensively been demonstrated to have significant antibacterial properties (more comprehensively reviewed by Dong *et al.*,^13^ Sánchez-López *et al.*^14^ and Zhang *et al.*^15^).

Given their size and selectivity for bacteria, metal-based nanoparticles (NPs) have proved to be highly effective against the pathogens^14^ listed as a priority by the World Health Organization. Among them, silver-based NPs represent maybe the most effective antibacterial agents in this class, while NPs carrying other metals (*i.e.*, gold, zinc, copper, *etc.*) have been observed to exert variable bactericidal activities.^14^

Graphene-based nanomaterials have been developed for many purposes spanning from the promotion of bacteria proliferation to microbial inhibition. These materials have been used as growth promoting agents of bacteria to accelerate interspecies electron transfer during anaerobic metabolism. On the other hand, graphene-based materials with antibacterial properties have been synthesized to prevent biofilm formation on membranes for water treatment, medical equipment, and tissue engineering scaffolds.^15^

Carbon dots, constituted by small carbon nanoparticle cores with adsorbed surface passivation molecules, are generally nontoxic. However, with their effective light-harvesting properties over a very broad spectral range from UV to near-IR, carbon dots have exhibited strong photodynamic antibacterial effects.^13^

**Fig. 1:**
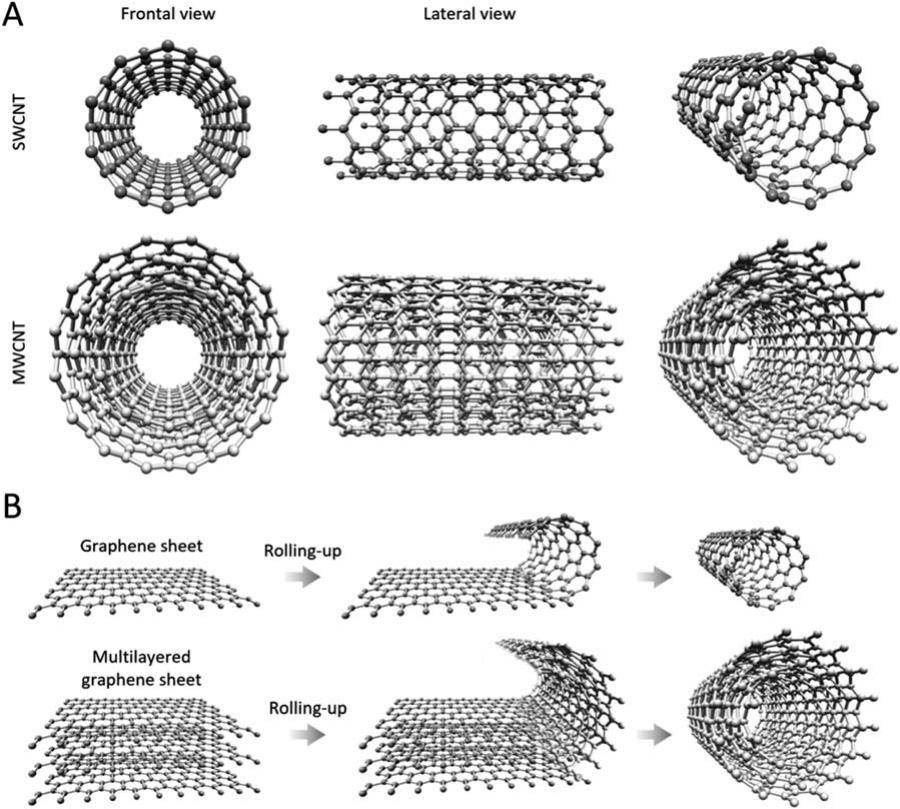
Comparison between SWCNTs and MWCNTs. (A) 3D representations of SWCNTs and MWCNTs. As a representative MWCNT, a triple-wallet CNT is shown. (B) Schematic representation of SWCNTs and MWCNTs generation. Image created with the Nanotube Modeler software (https://www.jcrystal.com/products/wincnt/).

Next to these nanomaterials, carbon nanotubes (CNTs) have also been demonstrated as highly efficient antibacterial agents over the past years. CNTs are cylinder-shaped allotropic forms of carbon, with diameters of several nanometres and lengths ranging from nanometres to tens of centimetres,^16–19^ depending on the targeted application and the employed synthesis protocols. CNTs originate from graphene sheets, whose layers appear as a rolled-up, continuous, hexagonal-like mesh structure, with the carbon molecules positioned at the apexes of the hexagonal structures (Fig. 1). CNTs with walls comprised of a single graphene sheet are known as single-walled carbon nanotubes (SWCNT) (Fig. 1A), while multi-walled carbon nanotubes (MWCNT) originate from the rolling up of several graphene layers^20^ (Fig. 1B). SWCNTs- and MWCNTs-based materials can be obtained by different preparation methods relying on chemical vapor deposition, laser ablation, flame synthesis, NP-assisted catalytic synthesis, and others,^20–22^ which results in important advantages, such as low-cost and wide-availability. A rich palette of protocols for functionalizing both SWCNTs and MWCNTs has been described in the literature to date,^23^ and among the various applications of obtained CNTs, their utility as therapeutic agents against MDR bacterial infections is generally acknowledged, holding great promise in the quest for next-generation antibacterial strategies that can lower antibiotic dosage or, in some cases, entirely replace the use of drugs.^24^ Importantly, in the context of fighting bacteria, CNTs have not been used only as antimicrobial agents but have also demonstrated important usefulness in sensing applications.^25,26^

Due to their great adaptability^10^ and excessive use of antibiotics^27^ over the years, microorganisms have developed different MDR phenotypes. In this focused review we discuss past efforts that were aimed at demonstrating the use of SWCNTs and MWCNTs as antimicrobial agents against *Staphylococcus aureus* (*S. aureus*) and *Escherichia coli* (*E. coli*), which have profiled over the past years as two prototypical Gram-positive and Gram-negative models. *S. aureus* is a Gram-positive bacterium, which causes a variety of mild to extremely severe infections, that is widely acknowledged as an important source for the spread of many antibiotic resistance genes worldwide.^28^*E. coli* is a species that is responsible for diverse pathological conditions with health hazards ranging from mild to severe,^29,30^ with drug-resistant *E. coli* strains posing a significant global threat.^31,32^ While antibiotic drugs remain the gold-standard in the fight against bacterial infections, the use of various nanomaterials as alternative solutions has been thoroughly explored in past studies.^33,34^ Most of these studies concluded that Gram-negative species are more resistant to membrane damages caused by nanomaterials than Gram-positive bacteria, given additional protection provided by their outer membrane.^35^ CNTs were as well considered in these past efforts, having been shown to be highly capable of severly damaging the cellular envelope (cell wall and membrane) leading to leakage of cytoplasmatic content and consequent cell death.^36^

While important work that has significantly contributed to the current level of understanding of the interactions taking place between CNTs and bacteria is also presented as background in the next section, we place the main emphasis on discussing selected works published in the past five years, that illustrate various facets on the use of SWCNTs and MWCNTs in antimicrobial applications. Considering that the use of these materials in their pristine form is known to present a series of limitations linked to aspects such as chemical inertness, hydrophobic character, poor adhesion during interaction with the cell wall, and instability in aqueous solution, we also focus our attention on SWCNTs and MWCTNs whose antibacterial properties were enhanced by the addition of functional groups *via* covalent and non-covalent bonds. In many cases, such strategies were found useful to reduce the dose of CNTs, and to achieve higher therapeutic efficacy compared to antibacterial solutions building on pristine CNTs. Overall, we consider this review to be a useful resource for those interested to get acquainted with the topic of antibacterial CNTs, and for peers interested in a glimpse on the current state-of-the-art.

## Fighting bacterial pathogens with SWCNTs: focused overview

### Pristine SWCNTs as antimicrobial agents

Despite convincing evidence reported to date on the antibacterial role of SWCNTs, a defined mechanism of action has not been univocally demonstrated so far.^24^ Many studies have attributed the antibacterial properties of SWCNTs to a wide range of potential mechanisms including metabolic alteration or inhibition,^37^ oxidative stress,^38^ and physical piercing damage to the cell envelope.^39^ Work performed to date has also shown that the antibacterial activity of CNTs can be influenced by: (i) structural properties (such as diameter, length, aggregation, and surface functional groups), (ii) concentration; (iii) buffer solution in which SWCNTs are solved; (iv) exposure time to SWCNTs, or (v) the number of collisions and extent of the impact forces occurring between the SWCNTs and the bacterial cells.^36^ With respect to the latter, in a landmark study reported by Kang *et al.*,^1^ it has been proposed that cell envelope damage, resulting from direct physical contact between bacteria and SWCNTs, represents in fact the major cause of bacterial death. The authors showed that SWCNTs exhibit stronger antibacterial activity than MWCNTs against *E. coli* K12, speculating that this probably relates to their smaller diameter size that facilitates partitioning and partial penetration into the cell envelope. However, the relationship between SWCNTs length and antimicrobial effects remains controversial: Aslan *et al.*^40^ demonstrated that the use of shorter SWCNTs caused additional damage to the surfaces of *E. coli* cells, which they hypothesized to be most likely related to the increased chances for interaction between the SWCNTs open ends and the targeted microorganisms. On the other hand, Yang and co-workers^41^ found that longer SWCNTs have stronger antimicrobial activity against the pathogenic bacterium *Salmonella typhimurium* due to their improved aggregation capability with bacterial cells, in a study that discussed as well limitations of short SWCNTs to bind to bacterial cells. With respect to other physical properties, Chen *et al.*,^42^ addressing in their work a broad-spectrum of CNTs, proposed that rigid and thin SWCNTs are more effective in terms of cell wall and membrane piercing of round-shaped bacteria than MWCNTs. An open debate still exists on what is the optimal CNT configuration for achieving the most efficient antibacterial effect.

Next, we discuss several relevant works, placing main focus on those published over the past five years, in which the antibacterial effects of SWCNTs, either in pristine form, or conjugated with other nanomaterials, or antibiotics, were discussed with respect to their activity on *S. aureus* and *E. coli* strains.

Among the wide palette of endeavours reporting SWCNT-based antibacterial tools, the work of Basiuk *et al.*^43^ investigated the antibacterial effects against *S. aureus* of pristine SWCNTs in comparison with nanodiamond graphene (ND) and graphene oxide (GO), two alternative carbonaceous nanomaterials that have gained increasing attention due to their presumed better biocompatibility compared to CNTs in specific scenarios, discussed in previous works.^44^ The authors found that among the tested nanomaterials, pristine GO exhibited the most pronounced antibacterial effects, exhibiting a dose-dependent behaviour. SWCNTs showed activity against *S. aureus*, but only at high concentrations (1 and 10 mg mL^−1^), while pristine ND was found not only to be less toxic but also to promote bacterial growth at the highest concentration assayed (10 mg mL^−1^). We consider this study to be important as it shows that carbonaceous nanomaterials exhibit consistently different antibacterial effects depending on their size and geometric configuration.

Noor *et al.*^39^ have addressed in their study the fact that SWCNTs are usually difficult to disperse, and thus many studies focusing on their antibacterial effects use them in combination with dispersion aides. While many of these contribute themselves to the exerted bacterial stress, many times such aspects are unaccounted for. The authors discussed thus the antibacterial effects of SWCNTs when administered with five dispersant agents: sodium dodecyl sulfate (SDS), Pluronic, lysozyme, DNA, and tryptic soy broth (TSB). They observed that SDS is fatal to *S. aureus* regardless of the presence of SWCNTs, while the activity of Pluronic and lysozyme against *S. aureus* was enhanced by the presence of SWCNTs. In contrast, DNA and TSB dispersions did not have any activity regardless of the presence of SWCNTs. Overall, the work of Noor *et al.*^39^ showed that studies focused on assessing the antibacterial activity of SWCNTs need to carefully consider the synergistic interactions taking place between these nanomaterials and dispersants, which may result in different levels of stress exerted on cells compared to the case when pristine SWCNTs are used without dispersion agents.

### Composite and functionalized SWCNTs as antimicrobial agents

The promising effects of pristine SWCNTs against bacterial pathogens prompted to the generation of functionalized forms of these CNTs to optimize their activity. In this body of efforts, Sah *et al.*^45^ have introduced as an efficient photodynamic antimicrobial chemotherapeutic agent a nano-composite made up of SWCNTs and amine-functionalized porphyrin. They showed that upon exposure to visible light, the porphyrin conjugated nanotubes inflict damage to *S. aureus* bacterial cells, finally leading to their death. Field emission scanning electron microscopy (FESEM) images of the cells treated with the photoactivatable nanocomposite showed the formation of web-like structures on the affected cells, which were dependent on the light irradiation time. This study showcases that the intrinsic antibacterial effects of SWCNTs can be significantly augmented by conjugating them with photosensitisers. In a different study addressing the use of SWCNTs in combination with photo-active nanomaterials, Mohammad *et al.*^46^ coated pristine SWCNTs with Ag-doped TiO_2_ NPs with a size estimated to range between 7.7 and 13.53 nm, which were found to be responsible for antibacterial photocatalytic effects. These nanocomposites were tested against bacterial strains of both *E. coli* and *S. aureus* models, with the authors observing Gram-negative bacteria to be more resistant to the proposed nanocomposite compared to Gram-positive bacteria, under illumination by UV light. MWCNTs were evaluated as well in this study, providing less efficient results. The authors speculate that although MWCNTs–TiO_2_/Ag showed a slightly lesser toxicity against bacteria in their experiments, in specific applications, they might represent the better choice given their more reduced-fabrication costs. Additional insights on CNTs based antibacterial phototherapies can be found in the thorough review work of Wang *et al.*^47^

**Fig. 2:**
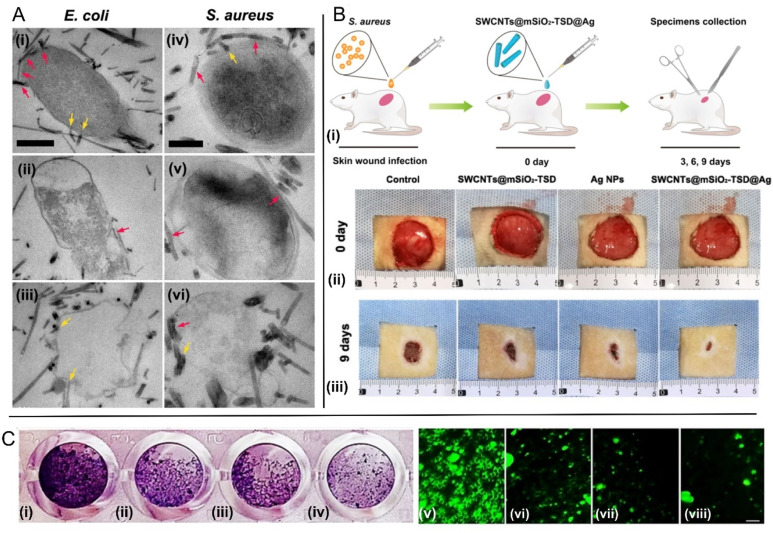
Fighting bacteria with SWCNTs. (A) TEM images of multi-drug-resistant bacteria *E. coli* (i)–(iii) and *S. aureus* (iv)–(vi) after treatment with SWCNTs@mSiO_2_-TSD@Ag. The SWCNTs@mSiO_2_-TSD@Ag that wrap around the bacteria and pierce into the cell walls are marked with red and yellow arrows, respectively. Scale bar, 500 nm (i)–(iii), 200 nm (iv)–(vi) [adapted with permission from Zhu *et al.*^48^]. (B) (i) Schematic diagram for the construction of rat skin wound infection model and the therapeutic process. Representative photos of cutaneous wounds in each group at 0 (ii), and 9 (iii) days after surgery [adapted with permission from Zhu *et al.*^48^]; (C) SWCNTs decorated with ZnO–Ag and ZnO–Au reduce *E. coli* biofilm formation. Left: quantification of biofilm formation as determined by crystal violet staining. Right: quantification of biofilm formation using fluorescence images collected using cells labeled with a nucleic acid stain. Scale bar: 10 μm. (i and v) Untreated control *E. coli*; (ii and vi) *E. coli* treated with functionalized SWCNTs; (iii and vii) *E. coli* treated with ZnO–Ag–SWCNTs; (iv and viii) *E. coli* treated with ZnO–Au–SWCNTs [adapted from Rugaie *et al.*,^55^ available under CC-BY license].

Considering other antibacterial routes, Zhu *et al.*^48^ introduced an ingenious antibacterial nanoplatform consisting of SWCNTs decorated with silver nanoparticles and coated with mesoporous silica. They showed that the outer mesoporous silica shells improve the dispersibility of SWCNTs, increasing their contact area with bacteria cell envelope, while the large number of mesopores in the silica layers act as microreactors for *in situ* synthesis of Ag NPs with controlled small size and uniform distribution. They compared the effects of this nanocomposite with the antibacterial properties of mesoporous silica coated SWCNTs and commercial Ag NPs, observing much stronger antibacterial performance against MDR *S. aureus* and *E. coli* strains, due to the larger extent of damage to the bacterial cell membranes, Fig. 2A, and the faster release of silver ions. Importantly, they also tested this nanocomposite *in vivo* using a rat skin wound infection model, showing remarkable bacterial clearance capabilities for MDR *S. aureus* strains, accompanied not only by great biocompatibility but also by valuable wound healing effects, Fig. 2B, which are known to be correlated with bacterial load.^49^

Besides past studies reporting the combination of SWCNTs with other nanomaterials to obtain more efficient therapeutic tools against bacteria, it is important to note that SWCNTs have also been successfully conjugated for this purpose with antibiotics. For example, Carver *et al.*^50^ proposed SWCNTs and nano-graphene oxide (NGO) as solutions for delivering the antibiotic tetracycline to a tetracycline-resistant *E. coli* strain. Tetracycline loaded-SWCNTs and NGOs were found to inhibit this strain, even for tetracycline amounts much lower compared to the minimum inhibitory concentration (MIC) of free tetracycline. This was attributed to the capacity of these two carbonaceous nanomaterials to transport the antibiotic into the cells and thus to circumvent the drug-resistance mechanism based on the expression of efflux pumps. SWCNTs were more efficient in delivering tetracycline compared to NGOs, which was attributed to their needle-like shape. This study consolidates the current belief that nanomaterials may represent a cornerstone for next-generation antibacterial therapies, showing that besides their intrinsic antibacterial properties, they can augment the effects of antibiotics, enabling their administration in lower doses, helping to reduce the selective pressure, and overcome antibiotic resistance.

In a study reported by Sapkota *et al.*^51^ the authors exploited for antibacterial purposes the fact that SWCNTs can be easily chemically combined with various semiconductor nanostructures such as ZnO, ZnS, SnO_2_, CdS or CuO. Considering the latter, they fabricated SWCNT–CuO nanocomposites by straightforward recrystallization accompanied by calcination, which resulted in heterojunctions being formed between the SWCNT surface and the CuO nanocrystals that were chemically attached to the SWCNT surface. Antimicrobial susceptibility assays demonstrated excellent bactericidal properties of the proposed material on both *E. coli* and *S. aureus* models. The authors attributed the bactericidal effects to the increased intracellular concentration of reactive oxygen species (ROS) resulted from the occurring chemical reactions, which are known to kill bacteria through cumulative oxidative stress.^52,53^ However, it is possible that endogenous ROS, produced by bacteria in response to membrane damage by SWCNT–CuO nanocomposites, may contribute to bacterial killing.^54^ Another study explicitly nominating ROS as the main antibacterial mechanism, is the work of Rugaie *et al.*,^55^ where the authors laced ZnO–Ag and ZnO–Au nanocomposites into SWCNTs to yield ZnO–Ag–SWCNTs, and ZnO–Au–SWCNTs. They showed that pre-treatment of phagocytic cells with these nano-hybrids activates these cells, enhancing phagocytosis and microbicidal activity by ROS and NADPH oxidase production. Moreover, this study demonstrated that ZnO–Ag–SWCNTs and ZnO–Au–SWCNTs nanocomposites contributed to the bactericidal activity against *E. coli* to a greater extent than the SWCNTs alone, Fig. 2C, as shown by the enhanced, excessive production of ROS, which is considered to be derived from increased NOX2 activation. This study thus highlights that SWCNT-based nanocomposites can stimulate the antibacterial response by the host innate immune system.

In a study addressing a different antibacterial function of SWCNTs, Kumar *et al.*^56^ functionalized pristine SWCNTs through acidic treatment for nucleation, followed by reduction of silver ions by microwave heating to produce Ag-NPs decorated SWCNTs (Ag–SWCNTs). *Via* a dip-dry-curing process, they coated on different cotton fabrics pristine SWCNTs, Ag-NPs and Ag–SWCNTs composites and qualitatively evaluated the antibacterial property of all coated fabrics against *S. aureus* and *E. coli*. The Ag–SWCNTs coated fabrics showed excellent antibacterial activity against both types of bacteria (the highest in the tested group), which did not significantly diminish even after many washings. This study represents an important example on the usefulness of SWCNTs-based nanocomposites to enable a next generation of fabrics that can prevent contamination/infection with bacteria, which can be especially useful in bacteria rich environments, such as hospitals.

Table 1 summarizes the main features of the described pristine and composite SWCNTs.

**Table 1:** Main features reported in the discussed studies addressing the antibacterial efficiency of SWCNTs

Typology	Average diameter^a^	Average length^a^	Solvent and/or dispersing agents^b^	Microorganism assayed	Activity	Reference
Pristine SWCNTs	2.8 nm	Variable (several μm)	Deionized water	*E. coli*	Reduction of bacterial viability; slow-down of metabolic activity; nucleic acids release	Kang *et al.*, 2008 (ref. 1)
Pristine SWCNTs	1.0 nm	300 nm	Chloroform and poly(dl-lactic-co-glycolic acid)	*E. coli*; *Staphylococcus epidermidis*	Membrane damages; slow-down of metabolic activity	Aslan *et al.*, 2010 (ref. 40)
Pristine SWCNTs	Not reported	Not reported	Isopropanol or distilled water	*S. aureus*; *E. coli*	Inhibition of *E. coli* growth (no activity on *S. aureus*)	Basiuk *et al.*, 2021 (ref. 43)
Pristine SWCNTs	1.25 nm	Three different lengths tested: <1.0 μm, 1.0–5.0 μm, and ∼5.0 μm	Deionized water	*S. typhimurium*	Reduction of bacterial growth; membrane damages	Yang *et al.*, 2010 (ref. 41)
Pristine SWCNTs	2.0 nm	1.0–5.0 μm	Tween-80	*Lactobacillus acidophilus*; *Bifidobacterium adolescentis*; *E. coli*; *Enterococcus faecalis*; *S. aureus*	Reduction of bacterial growth; loss of bacterial membrane potential; release of nucleic acids	Chen *et al.*, 2013 (ref. 42)
Pristine SWCNTs	0.84 nm	1.0 μm	Sodium dodecyl sulfate; Pluronic; lysozyme; DNA; tryptic soy broth	*S. aureus*; *S. typhimurium*	Inhibition of bacterial growth and viability	Noor *et al.*, 2022 (ref. 39)
SWCNTs–porphyrin conjugate	Not reported	Not reported	Ethanol	*S. aureus*	Inhibition of bacterial growth and viability	Sah *et al.*, 2018 (ref. 45)
SWCNTs–TiO_2_/Ag	1.0–4.0 nm	0.5–2.0 μm	Acidified distilled water	*S. aureus*; *E. coli*	Inhibition of bacterial growth and viability	Mohammad *et al.*, 2018 (ref. 46)
Silver nanoparticles-decorated-SWCNTs	20 nm	Not reported	Ethanol	*S. aureus*; *E. coli*	Inhibition of bacterial growth and viability; membrane damages	Zhu *et al.*, 2020 (ref. 48)
Mesoporous silica-coated-SWCNTs	20 nm	Not reported	Ethanol	*S. aureus*; *E. coli*	Inhibition of bacterial growth and viability; membrane damages	Zhu *et al.*, 2020 (ref. 48)
CuO-functionalized SWCNTs	2.27–16.67 nm	Not reported	Ethanol	*S. aureus*; *E. coli*	Inhibition of bacterial growth and viability	Sapkota *et al.*, 2020 (ref. 51)
SWCNTs decorated with ZnO–Ag NPs	30.0 to 65.0 nm	Not reported	Distilled water	*E. coli*	Moderate increase of bacterial killing by phagocytic cells; ROS production; inhibition of biofilm production	Al Rugaie *et al.*, 2022 (ref. 55)
SWCNTs decorated with ZnO–Au NPs	30.0 to 65.0 nm	Not reported	Distilled water	*E. coli*	Dramatic increase of bacterial killing by phagocytic cells; ROS production; inhibition of biofilm production	Al Rugaie *et al.*, 2022 (ref. 55)
Ag-NPs decorated SWCNTs	1.5 nm	5.0 μm	Distilled water	*E. coli*; *S. aureus*	Inhibition of bacterial growth	Kumar *et al.*, 2019 (ref. 56)

a The indicated average diameter and length are referred to SWCNTs after functionalization.

b Only solvent/dispersing agents used for antibacterial assays are indicated.

## Fighting bacterial pathogens with MWCNTs: focused overview

### Pristine MWCNTs as antimicrobial agents

MWCNTs are hollow cylindrical carbonaceous nanomaterials with walls composed of more than one sheet of graphene, with a typical diameter ranging in the order of tens of nanometres (Fig. 1). Among other significant roles that the nanotechnology community has identified for them, MWCNTs are regarded as promising tools in nanomedicine,^57^ with manyfold uses including as delivery vehicles for metallic nanoparticles, drugs, DNA aptamers, peptides, and proteins.^58,59^ Their intrinsic antibacterial properties are also widely acknowledged.^24^ Although it has been speculated that many of their cytotoxic effects in bacteria depend on multiple mechanisms, correlated with diverse factors such as amorphous carbon content, catalytic metal content, bundled conformation, length, and dispersity in aqueous media,^60^ the exact mechanisms behind their antibacterial modes of action are yet to be understood, mainly due to the scale at which MWCTNs–bacteria interactions take place, with many aspects unavailable to the resolution limitations of currently available characterization techniques. Similar to the mechanisms of actions acknowledged for SWCNTs, MWCNTs have been proposed to have membranolytic activities and/or to induce the production of ROS, which results in bacterial death due to massive oxidative stress.^52,53,61^ However, various studies proposed additional mechanisms over those of SWCNTs. For example, Mocan *et al.*^62^ suggested that the release of impurities upon MWCNT exfoliation may hold an important role in bacterial killing.

**Fig. 3:**
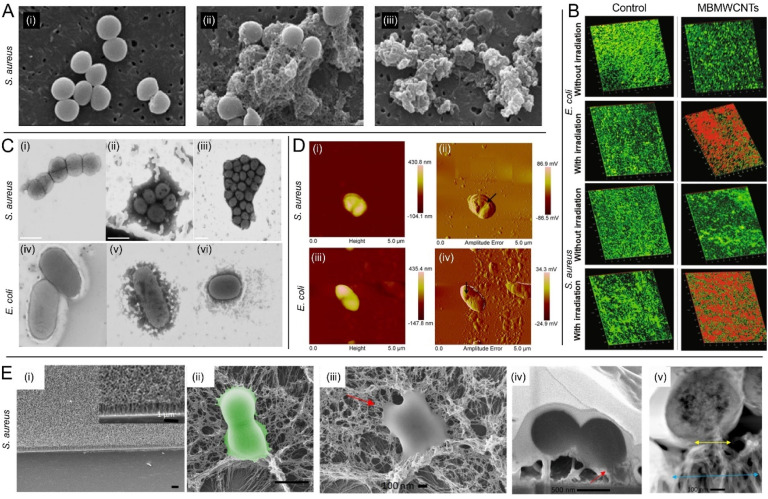
Fighting bacterial pathogens with MWCNTs. (A) SEM images of *S. aureus* at 80 000× magnification: (i) untreated control group, and microbial cells exposed to 100 μg mL^−1^ (ii) functionalized DWCNTs and (iii) functionalized MWCNTs [reproduced from Saleemi *et al.*,^63^ available under CC-BY license]. (B) Confocal microscopy 3D images of *E. coli* and *S. aureus* biofilms in the absence and presence of light irradiation. Biofilms treated with MBMWCNTs are displayed for comparison next to untreated biofilms in the control group. Red colour depicts dead cells [adapted with permission from Parasuraman *et al.*^68^]. (C) Scanning TEM images of (i) *S. aureus*; (ii) *S. aureus* + C1 (VCL/PEGDA–MNPs–GO–ZnMintPc); (iii) *S. aureus* + C2 (VCL/PEGDA–MNPs–MWCNTs–ZnMintPc); (iv) *E. coli*; (v) *E. coli* + C1; (vi) *E. coli* + C2 [adapted from Cuadrado *et al.*,^70^ available under CC-BY license]; (D) AFM images collected on *E. coli* and *S. aureus* after treatment with IL-1d@MWCNTs. (i and iii – AFM height; ii and iv-AFM amplitude error). The black arrows point to large holes in the bacterial cells, which may represent the mechanism by which cell death is achieved [adapted with permission from Bains *et al.*^76^]; (E) interaction of VAMWCNTs with *S. aureus* bacterial cells: (i) SEM images of a surface equipped with VAMWCNTs; scale bars 1 μm; (ii) false color SEM images of *S. aureus* revealing the bending of the MWCNTs on the functionalized surface and deformation of the bacterial cell membrane. Scale bars: 1 μm; (iii–v) biointerface of *S. aureus* and VAMWCNTs. (iii) Top-view SEM image of *S. aureus* showing altered cellular morphology due to the interaction with the VAMWCNT array. (iv) Focused ion beam-SEM image of *S. aureus* compromised by the flexible motion of MWCNTs leading to internalization of the MWCNTs and cell death; red arrows in (iii) and (iv): attachment of VAMWCNTs and stretching/loss of integrity of the bacterial membrane. (v) TEM micrographs showing a cross-sectional profile of the *S. aureus* cell. Blue and yellow arrows: Regions of affected MWCNTs due to contact with bacteria at the bottom, and at the top, respectively [adapted with permission from Linklater *et al.*^80^].

In a study reported by Saleemi *et al.*,^63^ it was shown that double-walled CNTs (DWCNTs) and MWCNTs inhibit the growth of many different opportunistic pathogens, including *S. aureus*, *P. aeruginosa*, *K. pneumoniae*, and fungal strains belonging to *Candida albicans*. Importantly, it was shown that the evaluated CNTs selectively damage the microbial cell walls or membranes, Fig. 3A, depending not only on the configuration of the nanotubes but also on the pathogen morphology. While studies focused on SWCNTs^40^ suggested that shorter CNTs are likely to induce more damage to bacteria, due to a higher chance of rupturing the envelope by the sharp ends, here it was hypothesized and partially demonstrated that longer CNTs may be more efficient as they wrap around the surface of the pathogen cell, yielding a higher surface contact area with the cell wall compared to shorter CNTs, with a proportional increase of efficacy. The authors also evaluated DWCNTs and MWCNTs dispersed by sodium dodecyl-benzenesulfonate (SDBS), which was used to improve the aqueous phase dispersion. FESEM images indicated strong interactions taking place between the SDBS-treated CNTs and the microbial cells, demonstrating also that stronger dispersion of CNTs increases their antimicrobial activity. Noteworthy, MWCNTs exhibited higher antimicrobial activity as compared to DWCNTs.

### Composite and functionalized MWCNTs as antimicrobial agents

MWNCTs usually exhibit moderate antibacterial properties compared to SWCNTs,^1,60^ thus prompting the development of various functionalization procedures to potentiate their activity. For example, Saleemi *et al.*^64^ investigated the antimicrobial role of thermoplastic polyurethane nanofibers containing various concentrations of surfactant-modified DWCNTs and MWCNTs against several Gram-positive and Gram-negative bacterial strains, including *S. aureus*. Different assays, such as number of viable cell count, diameter of inhibition zone, and growth curve values confirmed excellent microbicidal properties of the electrospun nanofibers.

In a different study reported by David *et al.*,^65^ MWCNTs decorated with ZnO, Ag and hydroxyapatite (Hap) NPs (with NP diameters ranging from 7 to 35 nm depending on their type) were shown to have a significant antimicrobial activity and to reduce biofilm formation by cells of *S. aureus*, *B. subtilis*, *P. aeruginosa*, *E. coli* and *C. albicans*. Among other observed advantages, all the decorated MWCNTs were found to exhibit a better dispersion in water, compared to pristine MWCNTs. Among the evaluated MWCNT instances, the highest antimicrobial activity (in terms of the largest diameter of inhibition zone) was observed for MWCNTs decorated with ZnO and Ag NPs. The biofilm formation assay also demonstrated that these two variants exhibit inhibition of biofilm formation, consolidating the idea that antimicrobial systems building on MWCNTs and Ag and ZnO NPs are valuable solutions to be considered in the fight against resistant pathogens and biofilm associated infections. In another study discussing the effects of MWCNTs on biofilms, Abo-Neima *et al.*,^66^ showed that MWCNTs functionalized *via* an interaction with nitric acid were able to prevent *E. coli* and *S. aureus* biofilm formation. Furthermore, these materials were found to be capable to disrupt mature biofilms leading to their detachment. Transmission electron microscopy images revealed morphological changes that reflect the damage mechanisms. The functionalized MWCNTs were found to biologically isolate the cells from their surrounding microenvironment, contributing to the development of toxic substances and placing the cells under oxidative stress, finally leading to their death. The antimicrobial and biofilm formation resistance properties of MWCNTs were also demonstrated in the study of Madenli^67^*et al.*, who studied MWCNTs blended polyethersulfone (PES) membranes, considering as model of target organisms *E. coli* and *P. aeruginosa*. Their results showed that, following the deposition of *E. coli* cells onto the membrane surface, no colonies were formed on composite membranes instances synthesized at particular MWCNT content levels, whereas for membranes of similar composition incubated in *P. aeruginosa* suspensions, consistently less biofilm formation occurred within 24 h. Importantly, the authors showed no MWCNT release during the water filtration of the composite membranes, which is important in light of potential applications for separation and purification.

As discussed also in the previous section, addressing SWCNTs, CNTs have a high potential to enable efficient antibacterial photodynamic therapies.^47^ In this context, Parasuraman *et al.*^68^ assessed an antimicrobial photodynamic therapy based on methylene blue-conjugated MWCNTs (MBCNTs) on biofilms of *E. coli* and *S. aureus*, Fig. 3B. Under illumination with a laser source emitting at 670 nm, biofilm inhibition, cell viability, and extracellular polymeric substances (EPS) reduction assays showed higher inhibition in *S. aureus* than in *E. coli.* This was found to be correlated with the fact that the binding and uptake of MBCNTs was greater in *S. aureus* compared to *E. coli*, which was consistent with previous work addressing the killing of Gram-positive and Gram-negative bacteria with nanoplatforms incorporating methylene blue.^69^ Another study evaluating an antibacterial photodynamic therapy based on MWCNTs has been performed by Cuadrado *et al.*^70^ They studied two magnetic nanocomposites based on GO and MWCNTs loaded with the photosensitiser menthol–zinc phthalocyanine (ZnMintPc). These were conjugated with iron magnetic nanoparticles and encapsulated in a lipophilic envelope, conferred by treatment with a biocompatible hydrogel based on *N*-vinylcaprolactam (VCL) and poly(ethylene glycol)diacrylate (PEGDA), used to help with the dispersion of the considered hydrophobic compounds in aqueous media. The two magnetic nanocomposites, VCL/PEGDA–MNPs–MWCNTs–ZnMintPc and VCL/PEGDA–MNPs–GO–ZnMintPc, were found to exhibit excellent photodynamic/photothermal effects under 630 nm illumination against *E. coli*, *S. aureus* (Fig. 3C), and *C. albicans*. While VCL/PEGDA–MNPs–GO–ZnMintPc nanocomposites were efficient only against *E. coli* and *S. aureus* the VCL/PEGDA–MNPs–MWCNTs–ZnMintPc instances were able to suppress all these three pathogens, demonstrating their broad-spectrum as antimicrobial agents building on photodynamic and photothermal effects. Considering recent progress reported on the topic of cancer cell killing *via* magneto-mechanical forces exerted by endocytosed magnetic nanoparticles,^71^ we have reason to believe that such strategies may soon become reality also in the context of antimicrobial applications. This may represent an important breakthrough given the complementarity of magneto-mechanical and photodynamic/photothermal therapies.^72^

In another relevant effort, Baek^73^*et al.* exploited the fact that metal oxides are known to increase mobility, surface area, and photocatalysis when combined with CNTs. Specifically, they evaluated the antibacterial effects of ZnO- and TiO_2_-conjugated MWCNTs and GO nanocomposites in relationship to *E. coli*. The ZnO-based nanocomposites exhibited a higher antibacterial role compared with the TiO_2_ based instances, with the authors obtaining antibacterial effects in terms of bacterial cell growth inhibition in the order ZnO–GO > ZnO–CNT > TiO_2_–GO > TiO_2_–CNT. This study also focused on identifying which of the four possible antibacterial mechanisms is mainly responsible for the observed antibacterial effects: (i) generation of ROS, (ii) physicochemical characteristics, (iii) the steric effect, and (iv) release of metal ions. ROS generation was found to be in lead over the others, with the physicochemical characteristics and the steric effect taking part of the contributing mechanisms as well. This study suggests also that GO-based nanocomposites are to be preferred over CNT-based nanocomposites, with Scanning Electron Microscopy (SEM) and Transmission Electron Microscopy (TEM) images revealing that GO-based nanocomposites exhibited better attachment to the bacterial surface, while CNT-based nanocomposites significantly aggregated to each other, diminishing thus the interaction chances with the cells. Another relevant study focused on ZnO–MWCNT nanocomposites has been reported by Shakir *et al.*,^74^ who evaluated Co doped-ZnO/MWCNTs nanocomposites synthesized by means of the sol–gel method. They evaluated various modifications occurring in the physical properties of instances synthesized under different Co doping concentrations. They observed the growth of spherical clusters over the surface of interlocking cylindrical tubes, and that the Co doped-ZnO/MWCNT hybrid nanocomposites exhibit high absorbance, and band gap narrowing upon increasing cobalt-doping concentration, which can facilitate a wide range of applications. With respect to the antibacterial effects, the authors observed high inhibition efficiency for instances synthesized under high concentrations of Co, for both *S. aureus* and *E. coli* models. Given that cobalt is known for good biocompatibility and low toxicity, the nanomaterials discussed in this study represent an interesting example on the synergy between CNTs and Co.

Same as SWCNTs, MWCNTs can also be used in combination with conventional antibiotic drugs. For example, Hassani *et al.*^75^ introduced a novel nano-drug synthesized by covalent grafting of modified MWCNTs with levofloxacin (LVX). The MWCNT–LVX agent was demonstrated to be highly efficient against *S. aureus* strains. The novel synthetic nano-drug possessed high loading capacity and pH-sensitive release profile *in vitro* and *in vivo*, exhibiting higher bactericidal activity in a mouse *S. aureus* burn wound infection model compared to the stand-alone use of LVX.^75^

In a different type of approach, Bains *et al.*,^76^ exploited the fact that ionic liquid (IL) and MWCNTs show significant synergistic effects given the occurring strong π–cation interactions.^77^ They developed a material based on IL-functionalized MWCNTs for hydrophobic coatings, showing their effectiveness over *S. aureus* (including a methicillin-resistant strain), and *E. coli*. By the help of atomic force microscopy (AFM) and SEM, they elucidated the mechanisms of action, Fig. 3D, which confirmed the motivation of their design which was selected given the hypothesized electrostatic interactions through the cationic moiety with the negatively charged bacterial membrane, and the cell enveloped damage potentially favoured by the considered hydrophobic carbon chain length. The proposed material was also evaluated as a coating on a PVC substrate, a scenario in which it exhibited remarkable inhibition of the bacterial cell growth *in vitro*. Overall, this study has great potential to favour the advent of next-generation antimicrobial surfaces with self-sterilizing abilities. Other important applications of CNTs in the context of antimicrobial surfaces are nicely presented in the recent review of Teixeira-Santos *et al.*^78^

Moskvitina *et al.*^79^ assayed different carbon-based nanomaterials and demonstrated that carboxyl-functionalized MWCNTs are endowed with a strong antibacterial potential against *E. coli* and *S. aureus* in terms of growth inhibition and alteration of membrane integrity, presenting an activity comparable to catalytic filamentous carbon with different orientations of graphene blocks, ionic carbon, and ultrafine explosive NDs.

Finally, we find noteworthy to highlight the mechano-bactericidal action of vertically aligned MWCNTs (VAMWCNTs), which was demonstrated in the landmark work of Linklater *et al.*^80^ In their study, the authors showed that VAMWCNTs arrays inactivate both Gram-negative (*P. aeruginosa*) and Gram-positive (*S. aureus*) bacterial cells. The mechanistic action leading to the bacterial cell death stems from the elasticity of the proposed nanostructures, exhibiting a high aspect ratio (100–3000) between their length (microns) and diameter (approximately 10 nm). The authors demonstrated that upon the adsorption of bacteria onto the nanostructured surface, the deflection and retraction of MWCNTs results in physical membrane perturbation and cell death (Fig. 3E). In the context of the current efforts devoted to developing antibacterial surfaces building on mechano-bactericidal effects,^81,82^ we argue that CNTs are likely to play an important role in the years to come for enabling such applications. Table 2 summarizes the main features of the described pristine and composite MWCNTs.

**Table 2:** Main features reported in the discussed studies addressing the antibacterial efficiency of MWCNTs

Typology	Average diameter^a^	Average length^a^	Solvent and/or dispersing agents^b^	Microorganism assayed	Activity	Reference
Pristine DWCNTs and MWCNTs	2.0–4.0 nm	10.0–20.0 μm	Sodium dodecylbenzene sulfonate solved in water	*S. aureus*; *P. aeruginosa*; *K. pneumoniae*; *C. albicans*	Reduction of microbial growth	Saleemi *et al.*, 2020 (ref. 63)
MWCNTs decorated with ZnO and Ag	Not reported	Not reported	Distilled water	*S. aureus*; *P. aeruginosa*; *E. coli*; *B. subtilis*; *C. albicans*	Inhibition of bacterial growth (no effects on *C. albicans*); biofilm eradication	David *et al.*, 2021 (ref. 65)
MWCNTs decorated with Hap	Not reported	Not reported	Distilled water	*S. aureus*; *P. aeruginosa*; *E. coli*; *B. subtilis*; *C. albicans*	Low inhibition of microbial growth; moderate biofilm eradication	David *et al.*, 2021 (ref. 65)
Nitric acid treated-MWCNTs	15.0 nm	2.0 μm	Ethanol	*E. coli*; *S. aureus*	Inhibition of bacterial growth and biofilm formation; biofilm disruption	Abo Neima *et al.*, 2020 (ref. 66)
MWCNT blended PES membranes	Not reported	Not reported	None	*E. coli*; *P. aeruginosa*	Inhibition of bacterial growth and biofilm formation	Madenli *et al.*, 2021 (ref. 67)
MBCNTs	50.0 nm	1.5 μm	Distilled water	*E. coli*; *S. aureus*	ROS content increasing; inhibition of bacterial growth and biofilm formation; protein leakage; lipid peroxidation	Parasuraman *et al.*, 2005 (ref. 68)
MWCNTs-magnetic nanocomposites	Not reported	Not reported	Tween 80 in distilled water	*E. coli*; *S. aureus*; *C. albicans*	Inhibition of microbial growth	Cuadrado *et al.*, 2022 (ref. 70)
ZnO- and TiO_2_-conjugated MWCNTs	Not reported	Not reported	Sulfuric acid and distilled water	*E. coli*	Inhibition of bacterial growth; increase of ROS content	Baek *et al.*, 2019 (ref. 73)
Co doped-ZnO/MWCNTs	8.0–15.0 nm	10.0–50.0 μm	Distilled water	*E. coli*; *S. aureus*	Inhibition of bacterial growth	Shakir *et al.*, 2021 (ref. 74)
LVX–MWCNTs	46.9 nm	10.0–30.0 μm	Distilled water	*S. aureus*	Inhibition of bacterial growth	Hassani *et al.*, 2022 (ref. 75)
IL–MWCNTs	100.0–200.0 nm	Not reported	None (dried PVC surface)	*E. coli*; *S. aureus*	Inhibition of bacterial growth; DNA-binding (role in bacterial growth inhibition unclear)	Bains *et al.*, 2020 (ref. 76)
VAMWCNTs	10.0 nm	Modulable depending on the growing time	None (dried surface)	*S. aureus*; *P. aeruginosa*	Alteration of membrane integrity	Linklater *et al.*, 2018 (ref. 80)
Carboxyl-functionalized MWNTs	Set of different MWCNTs with variable diameter and length		Distilled water	*E. coli*; *S. aureus*	Inhibition of bacterial growth; alteration of membrane integrity	Moskvitina *et al.*, 2023 (ref. 79)

a The indicated average diameter and length are referred to MWCNTs after functionalization.

b Only solvent/dispersing agents used for antibacterial assays are indicated.

## Brief considerations on current challenges in fighting antibiotic resistant strains with CNTs

**Fig. 4:**
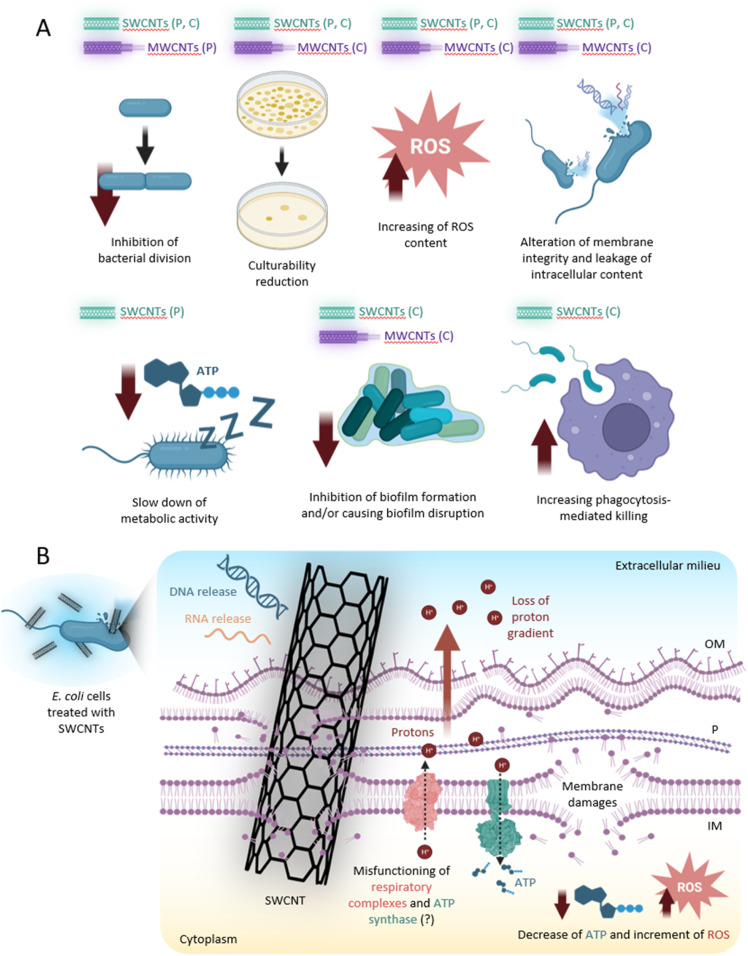
(A) Effects of SWCNTs and MWCNTs on bacterial cells. Letters P and C in brackets indicate that the displayed effects were documented on pristine and composite SWCNTs and MWCNTs, respectively. (B) CNTs putative mechanism(s) of action. CNTs cause alteration of membrane integrity by “stabbing” bacterial membranes, which results in (i) leakage of intracellular macromolecules (*e.g.*, nucleic acids), (ii) loss of proton gradient, and (iii) misfunctioning of the respiratory complexes and ATP synthase that (iv) determine a decrease of ATP production and an increment of ROS content. Abbreviations: OM, outer membrane; P, peptidoglycan; IM, inner membrane. Figure created with Biorender.

In Fig. 4 we display the main putative antibacterial mechanisms of action of CNTs, deduced from all the results discussed in this work. However, the absence of an unambiguous model raises the need for further light to be shed on the exact mechanisms caused at the molecular level by the upon bacteria interaction with CNTs. As the antibacterial mechanisms of CNTs seem to involve multiple cellular targets^23,24^ (*i.e.* cell wall, cell membrane, modifications of proteins and DNA by ROS-induced damages, *etc.*), it seems improbable that bacteria strains could develop resistance to these nanomaterials. However, to what extent a selection of resistant mutants could withstand the action of CNTs remains a puzzling question, which definitely warrants the requirement of further studies on the subject. However, it is important to note that bacteria could potentially develop significant morphological changes, which could impact the antibacterial efficiency of CNTs. Indeed, it seems that there is a strong relation between antibiotic resistance and MWCNT effect on *S. aureus* cells. The antimicrobial efficiency of MWCNTs seems to be lower in MDR strains, as compared to antibiotic-susceptible isolates. *S. aureus* strains resistant to antibiotics acting on cell wall are less susceptible to both pristine and functionalized MWCNTs.^83^ The idea that cell wall structural modifications could interfere with the antimicrobial activity of CNTs is also supported by the work of Hassani *et al.*,^75^ discussed in the previous section, which reports different antibacterial efficiency of the MWCNTs depending on the cell wall structure of the target cells.

The antibacterial activity of nanomaterials in highly organized multicellular communities is also a common challenge, as bacteria in biofilms behave completely different compared to their planktonic counterparts. Microbial biofilms are more tolerant to all known antimicrobials and host defence mechanisms; therefore, the management of biofilm-associated infections is challenging.^84^ A recent study^85^ showed that MWCNTs promote bacterial conjugative plasmid transfer in aqueous environment. The results of this study suggest that the presence of particular MWCNTs configurations, especially clustered, provide bacteria with novel surfaces for intense cell-to-cell interactions in biofilms and can promote bacterial horizontal gene transfer, hence potentially elevating the spread of antimicrobial resistance. This leads to the idea that results obtained in studies addressing planktonic bacteria cannot be straightforward extrapolated to applications addressing biofilms, therefore knowledge transfer between these two fields of research should be done with extreme caution.

## Conclusions

Due to the increasing number of drug-resistant bacterial strains and their high pressure on the sustainability of health system across the globe, there is an urgent need to reduce the exposure to antibiotics, which is known to favour the development of resistance mechanisms. To this end, nanomaterials have been widely explored to date as solution to replace antibiotics, or as means to enable lower antibiotic doses. In the frame of these efforts, CNTs have been found to hold important antibacterial potential. Knowledge on the mechanisms by which CNTs are capable to kill bacteria is constantly growing, together with the extent of functionalization routes that results in enhanced anti-microbial effects. In this focused review we have discussed recent progress on the use of SWCNTs and MWCNTs as the backbone of various antibacterial solutions and tools, placing main emphasis on works reported over the past five years. The discussed works showcase various ways by which SWCNTs and MWCNTs can contribute to overcoming the current crisis that humanity faces due to the emerging number of drug-resistant microorganisms. We hope that our work will inspire future research aimed at understanding in more detail the interactions taking place between CNTs and prokaryotic cells, and the advent of novel CNT-based “weapons” capable to efficiently fight drug-resistant and drug-susceptible bacteria.

## Author contributions

All authors identified relevant references and designed the structure of the manuscript. MA, ML, CC, AMH, PV and SGS wrote the initial draft. SGS, MA, ML and PV have prepared the figure artwork. ML and PV have prepared the tables. All authors read and revised the scientific content of the initial draft, and of the revised version, and helped on reorganizing text to promote clarity. SGS and PV coordinated the overall effort.

## Conflicts of interest

There are no conflicts to declare.

## Supplementary Material
